# The “Common Soil Hypothesis” Revisited—Risk Factors for Type 2 Diabetes and Cardiovascular Disease

**DOI:** 10.3390/metabo11100691

**Published:** 2021-10-09

**Authors:** Lilian Fernandes Silva, Jagadish Vangipurapu, Markku Laakso

**Affiliations:** Institute of Clinical Medicine, Internal Medicine, University of Eastern Finland, 70210 Kuopio, Finland; lilian.fernandes.silva@uef.fi (L.F.S.); jagadish.vangipurapu@uef.fi (J.V.)

**Keywords:** type 2 diabetes, cardiovascular disease, coronary artery disease, risk factors, Mendelian randomization, genetics

## Abstract

The prevalence and the incidence of type 2 diabetes (T2D), representing >90% of all cases of diabetes, are increasing rapidly worldwide. Identification of individuals at high risk of developing diabetes is of great importance, as early interventions might delay or even prevent full-blown disease. T2D is a complex disease caused by multiple genetic variants in interaction with lifestyle and environmental factors. Cardiovascular disease (CVD) is the major cause of morbidity and mortality. Detailed understanding of molecular mechanisms underlying in CVD events is still largely missing. Several risk factors are shared between T2D and CVD, including obesity, insulin resistance, dyslipidemia, and hyperglycemia. CVD can precede the development of T2D, and T2D is a major risk factor for CVD, suggesting that both conditions have common genetic and environmental antecedents and that they share “common soil”. We analyzed the relationship between the risk factors for T2D and CVD based on genetics and population-based studies with emphasis on Mendelian randomization studies.

## 1. Introduction

Type 2 diabetes (T2D) has reached epidemic proportions. According to the evaluation of the International Diabetes Federation, the number of individuals with diabetes will increase from 463 million adults in 2019 to 700 million by 2045 (https://www.idf.org/ access on 20 July2021) [1]. This increase is attributable mainly to T2D, which represents >90% of all cases of diabetes. Cardiovascular complications are the major cause of morbidity and mortality in people with T2D [2].

Cardiovascular disease (CVD) is the major cause of death globally, with an estimated 17.9 million death annually, that is 31% of all deaths (https://www.who.int/health-topics/cardiovascular-diseases/access on 20 July 2021) [3]. Coronary artery disease (CAD) and ischemic stroke are the most common manifestations of CVD. Major risk factors for CVD are male sex, high blood pressure, high low-density lipoprotein (LDL) cholesterol, smoking, and T2D [4].

CVD can precede the development of T2D, and T2D is a major risk factor for CVD complications, suggesting that these conditions share common genetic and environmental antecedents and spring from a “common soil” [5]. Indeed, a recent Mendelian randomization (MR) study concluded that genetic risk of T2D increased the risk of CAD in both women and men [6]. Additionally, several risk factors are shared between T2D and CVD, such as obesity, insulin resistance, dyslipidemia, and hyperglycemia [7]. Genome wide association studies (GWASs) indicated that CAD and stroke share partially genetic background with T2D, suggesting that genetic variants associated with an increased risk of CVD are also associated with an increased risk of T2D [8]. However, only a few loci jointly contribute to the risk of T2D and CVD [9]. One of the shared loci lies at chromosome 9p21.3 and includes *CDKN2A* and *CDKN2B* genes, identified in the earliest GWASs for T2D [10] and CAD [11]. The *IRS* locus and the genetic variants located within are also shared between T2D and associated traits, including insulin, triglycerides, obesity markers, and CAD [12]. The conclusion of the genetic studies on the relationship between T2D and CAD is that T2D causes CVD, but CVD does not cause T2D [13].

Even in a case where genetic loci are shared between T2D and CAD, this does not imply that mechanisms and pathways are identical in the risk of these diseases. Especially critical is to clarify the role of insulin resistance in CVD [14]. This is a very challenging task because there are no large populations where insulin resistance and insulin secretion were measured using the gold standard measurements, such as euglycemic clamp studies for insulin sensitivity and frequently sampled intravenous glucose tolerance tests for insulin secretion. Studies are largely missing where pathways for CVD and T2D are investigated. Shu and collaborators [15] identified the gene networks shared by both CVD and T2D. They reported that especially pathways regulating lipid and glucose metabolism are shared by CVD and T2D. Endothelial dysfunction and dysfunctional perivascular adipose tissue are observed in both atherosclerosis and T2D. Perivascular adipose tissue could be one of the mechanistic links between cardiovascular diseases and T2D [16].

The aim of this review was to examine the relationship between T2D and CVD by identifying risk factors and pathogenic pathways for these diseases. We especially emphasize evidence from the Mendelian randomization studies, allowing us to obtain causal conclusions.

**Figure 1:**
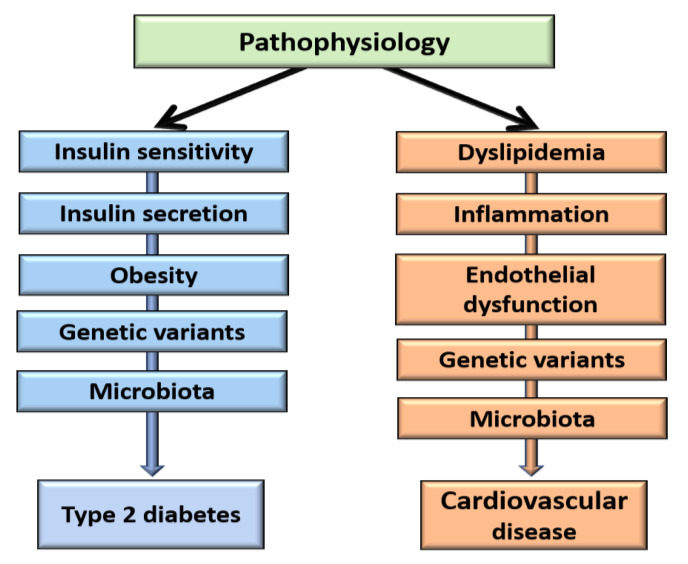
Pathophysiology of type 2 diabetes and cardiovascular disease.

The most important pathogenic pathways of T2D and CVD are presented in Figure 1.

## 2. Pathophysiology of Type 2 Diabetes

The main pathophysiological features in patients with T2D are impaired insulin secretion and insulin resistance in liver, skeletal muscle, and adipose tissue [2]. A long period of prediabetes often precedes T2D characterized by mild elevation of fasting or 2 h glucose levels [17]. Impaired pancreatic beta-cell function is also observed early in the development of T2D. Incretin hormone deficiency/resistance [18] and hyperglucagonemia [19] also contribute to disturbances in insulin secretion in T2D.

T2D is a polygenic disease. A Pro12Ala substitution of the *PPARgamma2* gene was the first replicated genetic variant associated with T2D [20]. There were 403 distinct association signals in T2D identified in the latest large meta-analysis of GWASs [8]. These signals explain close to 20% of the overall risk of T2D, and the polygenic risk score (PRS) including ~130,000 variants explained about 50% in analyses of the UK Biobank data [8]. Most of these variants in GWASs associated with T2D regulate insulin secretion, the most important pathophysiological abnormality resulting in the conversion to T2D.

Insulin resistance, a defect in insulin-mediated control of glucose metabolism in different tissues, prominently in muscle, adipose tissue, and liver, is one of the earliest manifestations of T2D and CVD. These diseases share intertwined metabolic abnormalities, including obesity, hyperinsulinemia, hyperglycemia, and hyperlipidemia [21]. In the insulin stimulated state, skeletal muscle is the major tissue responsible for glucose uptake. The liver maintains normal glucose levels by regulating gluconeogenesis and glycogenolysis. The main function of insulin in the adipose tissue is to prevent lipolysis [19]. Insulin resistance in different tissues is caused mainly by environmental and lifestyle factors, whereas only a few genetic variants for the risk of T2D identified in GWAS studies are associated with insulin resistance.

We generated PRSs including several genetic variants to evaluate changes in insulin secretion (PRS_IS_), insulin resistance (PRS_IR_), and incident T2D in the prospective METSIM study including a total of 76 genetic variants increasing the risk of T2D [22]. We found that PRS_IS_ was significantly associated with increased fasting glucose and incident T2D and decreased insulin secretion during the follow-up. However, PRS_IR_ failed to predict changes in glucose levels, or the conversion to diabetes. This finding supports the notion that decreased insulin secretion is more important than decreased insulin sensitivity for the conversion to T2D.

## 3. Pathophysiology of Cardiovascular Diseases

CVD includes CAD, myocardial infarction, and ischemic stroke. Ischemic CAD includes atherosclerosis coupled with thrombosis in coronary arteries leading to myocardial infarction. However, detailed understanding of molecular mechanisms leading to CVD events is still largely missing. Initiation of atherosclerosis involves three processes, atherogenic lipid deposition, pro-inflammatory condition, and endothelial dysfunction [23]. Recent advances in high-throughput technology including genomics, transcriptomes, proteomics, metabolomics, and metagenomics are likely to identify causal genes and reveal molecular mechanisms involved in CVD events [24].

Risks of CVD and T2D are determined by genetic and lifestyle factors. Several PRSs have been calculated which show an improvement in prognostic power when PRSs are added to existing CVD risk calculators. However, the contribution of PRSs for the risk of CVD events is substantially less than PRSs for T2D, and consistent results are not available regarding how much PRSs explain the risk of CVD events [25]. PRSs including thousands of genetic variants have about four-fold odds ratio for CAD when comparing individuals in the upper and the lower quintiles of the distributions of risk estimates [26].

The value of the PRSs is currently not obvious in clinical practice. Therefore, we need simple measurements for clinical practice which could help to identify individuals at high risk of both T2D and CVD. One possibility is the Finnish Diabetes Risk Score, which is composed of eight easily available parameters [27]. We found that the Finnish Diabetes Risk Score was significantly associated with an increased risk of incident T2D, CVD, and total mortality in 8.2 year follow-up of the METSIM cohort, including 8749 participants without T2D at baseline [28,29].

## 4. Microbiota in Type 2 Diabetes and Cardiovascular Disease

### 4.1. Type 2 Diabetes

The microbiota, consisting of various micro-organisms, mainly bacteria but also viruses, protozoa, and fungi, plays an important role in human health and disease [30]. The microbiota is responsible for food digestion, regulation of immune responses, and the synthesis of different metabolites resulting from microbial metabolic activities [31]. Imbalances or disturbances in the homeostasis between the microbiota and the host environment may play an important role in the pathogenesis of many disorders, including T2D and CVD [32]. Moreover, the microbiome produces metabolites that could be pathogenic or beneficial to the host. Previous studies suggested that patients with T2D [33] and CVD [34] have changes in the intestinal microbiota composition, although the results were conflicting.

We performed a 7.4 year follow-up study of the metabolic syndrome in men (METSIM) cohort including 5169 participants to investigate the role of microbiota-based metabolites as risk factors for T2D [35]. A total of 86 metabolites related to gut microbiota were included in statistical analysis. We found 12 metabolites that were significantly associated with increased risk of T2D. The following metabolites significantly increased the risk of T2D: creatine by 43%, palmitoleoylglycerol (16:1) by 41%, urate by 39%, 2-hydroxybuturate/2-hydroxyisobutyrate by 33%, kynurenate by 31%, xanthine by 31%, xanthurenate by 31%, 3-(4-hydroxyphelyl)lactate by 30%, 1-oleoylglycerol (18:1) by 29%, 1-myristoylglycerol by 23%, dimethylglycine by 20%, and 2-hydroxyhippurate by 19%. We also demonstrated that the conversion to T2D was associated with decreased insulin secretion during the follow-up, but the association with decreased insulin sensitivity for the conversion to T2D was not as strong. Interestingly, not all metabolites increasing the risk of T2D were associated with insulin secretion and insulin sensitivity, indicating that there may be other lesser known mechanisms increasing the risk of T2D.

We failed to find a significant association between trimethylamine N-oxide (TMAO) and incident T2D. However, a recent meta-analysis based on cross-sectional data reported a significant association between circulating TMAO levels and increased the risk of diabetes [36]. Our measurements did not include short-chain fatty acids (SCFAs), acetate, propionate, or butyrate, which have been linked to T2D. SCFAs regulate host glucose homeostasis by stimulating the secretion of peptide YY and GLP-1 [37].

Sanna et al. included in their study 952 participants with normal glucose. These individuals had data available on genome-wide genotyping, gut metagenomic sequencing, fecal SCFA levels, and genome-wide association summary statistics for metabolic and anthropometric traits [38]. The authors found in their MR analysis that host genetic-driven increase in gut production of the SCFA butyrate was associated with improved insulin secretion. They also reported that abnormalities in production or absorption of the SCFA propionate were causally related to increased risk of T2D. This study gives important evidence that the gut microbiome is causally related to metabolic traits.

### 4.2. Cardiovascular Disease

Several studies suggest that the intestinal microbiome is linked to the risk of CVD. Microbiome composition and diversity changes have been found in patients with atherosclerosis and in individuals with several CVD risk factors, including hypertension, dyslipidemia, adiposity, glucose homeostasis, vascular inflammation, and insulin resistance [39,40].

Associations of gut microbiota-derived metabolites, including TMAO, SCFAs, and bile acids with CAD, were published in several metabolomics studies [41]. The first studies revealing a potential causal link between the gut microbiome and CVD were focused especially on TMAO [41]. Large-scale cohorts reported associations of TMAO levels with CAD and stroke [42,43]. However, not all studies observed the relationship between increased TMAO levels and incident CVD. Jia et al. [44] reported that genetically predicted higher TMAO and carnitine were not significantly associated with increased risk of CAD, myocardial infarction, or stroke.

Gut microbiota may also play an important role in the development of heart failure that is often a consequence of a CAD event. Metabolites such as SCFA, TMAO, amino acid metabolites, and bile acids reflect complex host–microbe interactions in heart failure [45]. However, the evidence on the relationship between altered gut microbiota and heart failure is mostly based on preclinical studies, and, therefore, clinical studies are needed to confirm the causality of gut microbiota on the risk of heart failure.

The studies on microbiota have been performed almost entirely in European/White Caucasians, which is a limitation since compositional differences in microbiota between populations and genders may translate into differences in susceptibility to different disease, including T2D and cardiovascular disease.

## 5. Approaches to Identify Risk Factors for Type 2 Diabetes and Cardiovascular Disease

**Figure 2:**
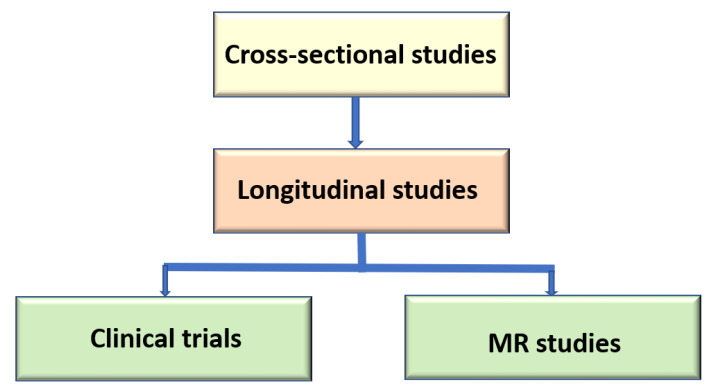
Different types of studies for the identification of risk factors for type 2 diabetes and cardiovascular diseases (MR, Mendelian randomization study).

Several approaches have been applied to identify risk factors for T2D and CVD (Figure 2). Cross-sectional studies are only hypothesis generating studies. Longitudinal population studies are needed to test if the risk factor predicts T2D or CVD. Causality of a risk factor for a given disease is tested by the Mendelian randomization (MR) approach.

### 5.1. Population-Based Studies

Finding early markers for chronic diseases, including T2D and CVD, is important for diagnosis, prediction, prognosis, and treatment of these diseases. Especially important is to find individuals who are at high risk for T2D and/or CVD given the fact that these diseases are very frequent worldwide and lead to long-term complications and mortality. Several cross-sectional studies have been published to find early markers for the risk of T2D and CVD. Often, these studies have been small, not representative of the background population, and reported conflicting findings compared to other studies [46]. Large longitudinal population-based cohort studies give more reliable results.

An example of a longitudinal population-based cohort is the METSIM study, which includes 10,197 men having a follow-up currently over 10 years [43]. Diagnosis of T2D is based on the ADA criteria [47]. We found, in the follow-up study of the METSIM cohort, that multiple laboratory measurements and metabolites were significantly associated with the risk of T2D [48]. In a recent study, we had metabolomics data for all 20 amino acids in 5181 participants of the METSIM study. Nine amino acids were significantly associated with decreases in insulin secretion (disposition index) and increases in glucose levels. Of these amino acids, tyrosine, isoleucine, alanine, aspartate, and glutamate were also significantly associated with an increased risk of incident T2D [49]. Our study was a hypothesis generating study because previous studies have not reported that amino acids are associated with decreases in insulin secretion.

Meta-analyses of several cohorts give often only limited additional information about the significance of different risk factors for T2D because of the heterogeneity of different studies. A recent review included 86 meta-analyses and MR studies. Only 46 of 142 associations were statistically significant at the level of *p* < 10^−6^ [50]. Although association studies may have importance for the understanding of potential risk markers for T2D, they do not prove causality. We reported several statistically significant associations from the METSIM follow-up study; the most significant was the association of mannose with T2D (hazard ratio, HR, 1.80, 95% confidence intervals, CI, 1.32–2.27) [51]. Mannose correlates significantly with glucose concentrations [52] and is associated with decreased insulin sensitivity and insulin secretion. Mechanisms related to how mannose increases the risk of T2D remain unknown. Recently, an MR study confirmed that mannose is causally associated with T2D [53].

### 5.2. Mendelian Randomization Studies

Population-based studies have limitations since they report only associations of risk factors with the disease of interest. Understanding the difference between association and causation is crucial when trying to reveal disease mechanisms [54].

**Figure 3:**
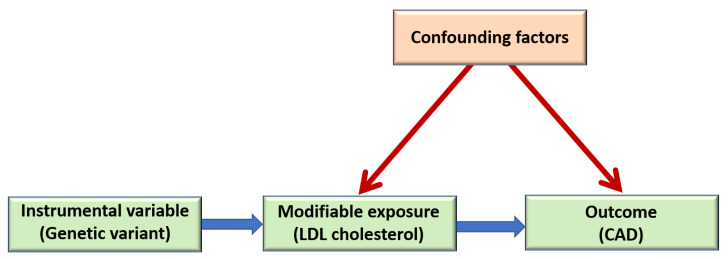
Mendelian randomization study. Genetic variants or polygenic risk scores are instrumental variables, for example, LDL cholesterol is a modifiable exposure, and coronary artery disease (CAD) is an outcome.

MR studies can assess causality (Figure 3). The risk factor is represented by genetic variant(s) for that risk factor (instrumental variables). Genetic variants for the risk factor are associated with the outcome if the risk factor is causal. Instrumental variable could be one genetic variant or multiple genetic variants (PRS). Genetic variants are proxy measures for exposures (e.g., biomarkers, clinical measurements) and thus are free from reverse causation and confounding factors [55].

The traditional method to prove causality is the randomized controlled trial measuring the effects of an intervention on the risk of disease. Randomized controlled trials permit causal inference because they include random allocation to intervention group(s), blinding, which avoids bias, and intervention with prospective follow-up, which avoids reverse causation [55]. MR studies simulate clinical trials where cases and controls differ only with respect to the risk factor, but the key advantage over clinical trials is that genetic variants have a lifelong effect, whereas, in clinical trials, the exposure lasts only months or years. The validity of the genetic variants as instrumental variables is crucial for the reliability of an MR study. A valid instrumental variable should not be associated with confounders of the risk factor–outcome association. Furthermore, genetic variants should not have pleiotropy, meaning that a variant cannot affect risk factors on different causal pathways [56]. The recent GWASs reported that pleiotropy is more common than previously thought, since one variant can regulate tens of phonotypes. A further limitation of MR studies is population stratification. Calculation of the magnitude of the causal effect requires linearity of the relationships [57]. Given the abundance of GWAS studies, we can quite easily find information about pleiotropy of genetic variants of interest and test the presence of linkage disequilibrium. The lack of knowledge about confounding factors is often an important limitation of MR studies. Therefore, the results from MR studies should be evaluated critically, especially with respect to clinical implications.

## 6. Is Type 2 Diabetes a Causal Risk Factor for Cardiovascular Disease?

Several large MR studies were published that support T2D as a causal risk factor for CVD [13,58,59,60] and ischemic stroke [60,61]. Odds ratio (OR) for CAD in these studies varied from 1.11 to 2.35 and OR for ischemic stroke from 1.13 to 1.42. In MR studies, fasting and non-fasting glucose and HbA1c were also associated with CAD [13], supporting the role of hyperglycemia as a causal risk factor for CAD.

MR studies also showed that genetically predicted fasting insulin is a causal risk marker for CAD [62,63] and myocardial infarction [64]. Similarly, genetically predicted insulin secretion was shown to predict CAD (OR 1.83, 95% CI, 1.19–2.62) as well as insulin resistance (OR, 2.35, 95% CI, 1.46–3.53) [65]. However, elevated fasting insulin level is not a reliable marker for insulin resistance since it reflects not only insulin resistance but also insulin secretion and insulin clearance by the liver [66].

## 7. Risk Factors for Type 2 Diabetes and Cardiovascular Disease: Evidence from Mendelian Randomization Studies

### 7.1. Type 2 Diabetes

T2D has two major pathophysiological mechanisms, impaired insulin secretion from the pancreas and insulin resistance, especially in skeletal muscle and liver [67]. Interplay between genetic factors and environment is needed for the development of T2D. Furthermore, not all disease-causing pathways leading to this disease are completely understood. A total of 403 distinct association signals have been reported, explaining 18% of the risk of T2D and offering insights into biological pathways causal for T2D [8]. Most of the genetic variants whose functions are known regulate insulin secretion and a minority regulate insulin sensitivity. Age, obesity and its distribution, behavioral factors (physical activity, smoking, diet), family history of diabetes, hypertension, lipid disorders, different drug treatments, etc., were reported to be risk factors for T2D [67]. However, the causality of these risk factors needs to be evaluated by MR studies.

Table 1 lists MR studies reporting causality of risk factors for T2D and CVD. The study by Wang and collaborators [68] included participants with and without T2D from the Nurses’ Health Study and the Health Professionals Follow-Up Study. This MR study included five genetic variants associated with low birthweight and reported a 2.94-fold increased risk for T2D, suggesting that intrauterine exposures are important in the pathogenesis of T2D.

Increased BMI is the driving force for the epidemic of T2D. The study by Corbin et al. [70] included 96 genetic variants and 12,171 cases with T2D and 56,862 controls of mainly European descent. They reported that BMI was causally related to a 26% increased risk for T2D, and waist-to-hip ratio adjusted for BMI was related to an 82% increased risk for T2D. These observations agree with previously published population-based studies showing that waist-to-hip ratio is an independent risk factor for T2D [72]. Within low- and middle-income areas such as Sub-Saharan Africa, the burden of diabetes is already significantly driven by many factors, including socioeconomic, nutritional (high-calorie “western diet”, obesity), and lifestyle (physical inactivity) changes. However, in this population, T2D and CVD can also occur in people with low BMI [80].

Epigenetics changes result in altered gene function without a change in the DNA sequence. Aging, genetic predisposition, and several environmental factors, including exercise and diet, contribute to epigenetic variability in type 2 diabetes and obesity [81]. Wang et al. [82] reported a link between obesity, DNA methylation, and gene expression for several genes in African American youth and young adults. An example from cardiovascular disease is angiogenic long non-coding RNAs and their epigenetic regulation of the vascular endothelium [83].

Aikens et al. [73] included 28 genetic variants associated with systolic blood pressure to investigate their impact on the risk of T2D in European-centric meta-analysis including 37,293 cases and 125,686 control subjects. In their study, an increase in systolic blood pressure levels by 1 mmHg was associated with a 2% increase in the risk of T2D. Yuan et al. [75] included summary-level data for T2D from a meta-analysis of 32 GWASs including 898,130 individuals of European ancestry and 377 genetic variants significantly associated with smoking. Genetically predicted smoking was associated with a 28% increased risk of T2D.

Based on UK Biobank data, Zanetti et al. [77] measured several circulating biomarkers for T2D (33,323 cases and 418,610 non-cases). LDL cholesterol had a non-significant reduction in the risk of T2D (OR 0.82, 95% CI, 0.63–1.00). Total triglycerides increased the risk of T2D by 35%. ALT, a marker of fat in the liver, increased the risk of T2D by 73%. However, the association of CPR was not significantly associated with an increase in the risk of T2D. MR studies on the association of interleukin 6 (IL-6) with T2D are not available.

### 7.2. Cardiovascular Disease

Zanetti et al. [69] investigated the association of birthweight with CVD in 237,631 individuals from the UK Biobank (Table 1). A total of 5542 had CVD events, and 2656 had CAD events at baseline. High birthweight was associated with a 31% reduction in the risk of CAD. Gill et al. [71] reported that BMI increased the risk of CAD by 49% and waist-to-hip ratio by 54% based on consortia and UK Biobank genetic association summary data from 60,801 cases and 123,504 controls who were predominantly of European ancestry.

In an MR study by Malik et al. [74] including 255,714 European ancestry participants without a history of CVD, the risk of incident CVD increased by 49%, CAD by 50%, and stroke by 44% for every 10 mm Hg increase in genetically proxied systolic blood pressure. An MR study by Levin el al. [76] based on summary statistics from GWASs of smoking (60,801 cases and 123,504 controls) reported that smoking was associated with increases by 48% in CAD and by 40% in stroke.

Zanetti et al. [77] reported several circulating biomarkers for CAD (9647 cases and 451,933 non-cases) based on the UK Biobank data. LDL cholesterol increased the risk of CAD by 44%, total triglycerides increased the risk of CAD by 31%, and alanine transaminase (*ALT*), a marker of fat in the liver, increased the risk of CAD by 45%. IL-6 increased the risk of CAD by 60% in a meta-analysis of 19 studies including 9417 CAD patients and 15,982 controls. CRP did not significantly increase the risk of CAD.

Niu et al. [78] reported in their MR study including meta-analysis of 19 studies (9417 CAD patients and 15,982 controls) that the association between circulating IL-6 levels and the risk for CAD is causal using a genetic variant G-174C of the *IL6* gene as an instrument. One pg/mL elevation in IL-6 levels was associated with a significant increase in the risk of CAD (OR 1.60, 95% CI, 1.44–1.72, *p* < 0.01).

Richardson et al. [79] obtained data from CARDIoGRAMplusC4D (60,801 cases and 123,504 controls) and performed univariable and multivariable MR analyses for the association of apolipoprotein B with CAD. In multivariate MR analysis, apolipoprotein B was significantly associated with the risk of CAD (OR 1.92, 95% CI, 1.31–2.81, *p* < 0.001).

### 7.3. Mendelian Randomization Studies in Type 2 Diabetes and Cardiovascular Disease: Are There Differences?

A review of MR studies indicates that there are no major differences in the risk factors for T2D and CVD. Low/high birth weight, BMI, waist-to-hip ratio, blood pressure, smoking, total triglycerides, and ALT quite similarly increased the risk of T2D and CVD. However, there was a difference in LDL cholesterol concentration between T2D and CVD. T2D was associated with low levels and CVD with high levels of LDL cholesterol. Additionally, the markers of low-grade inflammation seem to be more important for the risk of CVD than for the risk of T2D.

Several studies reported that treatment with statin lowered LDL cholesterol and increased the risk of T2D [84,85], indicating that a low LDL cholesterol concentration seems to increase the risk of T2D. Swerdlow et al. [86] assessed in their MR study whether this increase in the risk of T2D is caused by inhibition of HMGCR. The authors included in their analysis genetic variants of the 3-hydroxy-3-methylglutaryl-CoA reductase gene (*HMGCR*). Their study showed that genetic variants of *HMGCR* that lowered LDL cholesterol in 129,170 individuals from randomized trials increased the risk of T2D (OR 1.12, 95% CI, 1.06–1.18). Therefore, an increased risk of T2D in individuals on statin treatment is at least partially related to HMGCR inhibition. Importantly, these investigators also reported increases in body weight, waist circumference, fasting insulin, and glucose concentrations. These observations agree with our study [75] showing that statin treatment increases insulin resistance and impairs insulin secretion. Further genetic evidence is the observation that T2D is less prevalent in individuals with familial hypercholesterolemia having elevated LDL cholesterol concentration compared to those who do not have this disease [87].

Low-grade inflammation has an important role in T2D and especially in CVD. Inflammatory processes in the arterial wall driven by modified lipoproteins trigger hypercoagulable state [88,89]. The effects of anti-inflammatory drugs to reduce CVD events is likely explained by their interaction with the pathway of the NLRP3 inflammasome to interleukin 1, IL-6, and CRP [90]. Moreover, clinical trials showed that the pharmacological inhibition of several pathways involved in inflammation are beneficial among patients with manifest CVD events. The CANTOS trial recruited 10,061 patients with previously diagnosed myocardial infarction and increased CRP levels. In that trial, canakinumab monoclonal antibody against IL-1beta significantly reduced concentrations of IL-6 and high sensitivity CRP by 40%–60% as well as CVD events [91]. The COLCOT trial showed that early initiation of colchicine therapy reduced CVD events after myocardial infarction [92].

MR studies have linked low-grade inflammation to CVD events but not to T2D. However, this does not exclude the possibility that low-grade inflammation plays an important role in the risk of T2D.

## 8. Mendelian Randomization Studies on Metabolites in Type 2 Diabetes and Cardiovascular Disease

Recent developments in metabolomics have provided tools to investigate shared pathways between CVD and T2D. This method makes it possible to characterize changes in metabolites associated with different diseases and to identify early markers for diseases of interest. Mass spectrometry with liquid or gas chromatography has high sensitivity and resolution and allows detection and quantification of thousands of metabolites [93]. The high-throughput serum nuclear magnetic resonance platform is another method to quantify metabolites. However, the accuracy of this method is substantially lower compared with mass spectrometry, and the number of metabolites identified using this method is low compared to mass spectrometry [94].

The metabolomics approach has limitations. There is no consensus on how metabolomics results are standardized, and protocols, statistical approaches, as well as instrumentation may differ, which could yield varying sets of metabolites [95]. Furthermore, metabolomics studies including large populations are expensive.

Most of the metabolite studies published thus far focused on T2D, with only a few on CVD. High branched chain amino acid levels were associated with T2D and insulin resistance in previous population-based studies [96]. The strongest signal among branched chain amino acids in a GWAS was for leucine encoding the rate-limiting step in branched chain amino acid catabolism [97]. The authors reported that a genetically predicted difference of 1 SD in leucine level was associated with an OR of 1.85 (95% CI, 1.41–2.42, *p* = 7.3 × 10^−6^) for T2D and claimed that leucine has a causal role in the etiology of T2D.

Wang Q et al. [98] included in their study 53 genetic variants associated with insulin resistance from a previous GWAS to explore their effects on 58 circulating metabolites. Their study provided genetic evidence that insulin resistance has a causal role on branched chain amino acids, in contrast to a previous study [97]. The authors concluded that their findings support the hypothesis that the metabolism of branched chain amino acids may be a mediator that is downstream of insulin resistance on the causal pathway to T2D [98].

Merino et al. [99] identified metabolites associated with the risk of T2D in a population-based prospective study including 1150 participants from the Framingham Heart Study Offspring. In adjusted Cox proportional hazard analyses, the risk of T2D per 1 SD increase in glycine was 0.65 (95% CI, 0.54–0.78), in taurine 0.73 (95% CI, 0.59–0.90) and in phenylalanine was 1.35 (95% CI, 1.11–1.65). MR analysis demonstrated that the risk of T2D per 1 SD genetically increased glycine was OR 0.89, 95% CI, 0.80–0.99 and for phenylalanine was OR 1.6, 95% CI, 1.08–2.4.

Jia et al. [41] used several previously published databases for disease outcomes, CAD (60,801 patients and 123,504 controls), myocardial infarction (43,676 patients and 128,197 controls), and stroke (37,792 patients and 397,209 controls). Genetically predicted higher TMAO was not associated with an increased risk of T2DM, CAD, myocardial infarction, or stroke. However, they observed that genetically increased choline was suggestively associated with higher risk of T2D per 10 units (1.84, 95% CI, 1.00–3.42, *p* = 0.05), and betaine was associated with lower risk of T2D per 10 units (0.68, 95% CI, 0.48–0.95, *p* = 0.023).

Feofanova et al. [100] conducted GWAS on 640 circulating metabolites in 3926 Hispanic Community Health Study/Study of Latinos participants and replicated the results in 1509 participants in the ARIC study. In their MR study, the novel findings were that 1 SD increase in 1-arachidonylglycerol (20:4) (OR 1.13, 95% CI, 1.06–1.21) and 1-palmitoyl-2-stearoyl-GPC (16:0/18:0) (OR 1.24, 95% CI, 1.10–1.40) increased the risk of T2D and that 1 SD increase in gamma-CEHC (OR 0.84, 95% CI 0.78–0.91), gamma-CEHC glucuronide (OR 0.77, 95% CI, 0.67–0.87), and octadecanedioate (OR 0.81, 95% CI, 0.81–0.90) decreased the risk of CAD.

A study by Yun et al. [101] included 826 men and 1148 women without T2D from Beijing and Shanghai. They found that 11 novel and three reported sphingolipids were positively associated with incident T2D (OR 1.14–1.21, all *p* < 0.001) after multivariate adjustment including lifestyle characteristics and BMI. In their MR analysis, only ceramide (d18:1/20:1) was causal for the risk of T2D (OR 1.15, 95% CI, 1.05–1.26, *p* = 0.002). This study demonstrates that, from several statistically significant metabolite associations, only a few are causal.

## 9. Towards “Precision Medicine”

Both T2D and CVD are heterogeneous diseases. T2D is defined by increased fasting, 2 h glucose concentrations, or elevated hemoglobin A1c. CVD includes several pathways and mechanisms and therefore does not have an exact definition compared to T2D. Additionally, the genetic basis of CVD does not currently allow sub-classification of all manifestations of CVD.

Impaired insulin secretion and insulin resistance are the two major mechanisms in the pathophysiology of T2D. However, many mechanisms resulting in hyperglycemia are still poorly known. Recent progress in the genetics of T2D helps to identify subgroups in T2D. Dimas et al. [102] examined the associations of 37 established T2D susceptibility loci with indices of proinsulin processing, insulin secretion, and insulin sensitivity in 58,614 individuals without diabetes and found five clusters of the risk variants for T2D. The first cluster included four susceptibility loci having effects on insulin sensitivity; the second cluster had two susceptibility loci having effects on decreased insulin secretion and fasting hyperglycemia; the third cluster had one susceptibility locus having effects on insulin processing; the fourth cluster included five susceptibility loci having effects on insulin processing and secretion; and the fifth cluster included 20 susceptibility loci with no clear-cut associations with glycemic traits [94].

We applied Bayesian non-negative matrix factorization clustering analysis to find new clusters based on 94 T2D genetic variants and 47 diabetes-related traits [103] and found five clusters of T2D loci and traits. The first two clusters were associated with reduced insulin secretion, and three clusters were associated with insulin resistance. Importantly, genetic risk scores in these clusters were associated with different clinical outcomes, including CAD, stroke, and hypertension [103]. The approach of Ahlqvist et al. was different from our approach [104,105]. In their study, individuals were clustered based on six clinical and laboratory parameters, and genetic variants were not used in the identification of the clusters, which is a limitation of their study.

Wagner et al. [106] identified subgroups for T2D by using partitioning on variables derived from an oral glucose tolerance test, body fat distribution, liver fat content, and genetic risk in a cohort of extensively phenotyped individuals at increased risk for T2D. They identified six distinct clusters of sub-phenotypes. Three of these had increased glucose levels, but only individuals in clusters five and three had an increased risk of T2D. Individuals in cluster six had an increased risk of kidney disease. This study demonstrates heterogeneity in the pathophysiology in the prediabetes and identifies individuals with prediabetes who develop complications without rapid progression to overt T2D.

Many challenges remain to be solved before “type 2 diabetes precision medicine” will be applied in clinical practice. However, recent progress in studies identifying separate subgroups of patients with T2D is promising, because it likely helps to identify subgroups of patients having different pathophysiology and treatment.

## 10. “Common Soil” Hypothesis—Does It Apply to T2D and CVD?

Dr. Stern introduced his “common soil” hypothesis in 1995 by stating, “Unlike classical microvascular complications, large-vessel atherosclerosis can precede the development of diabetes, suggesting that rather than atherosclerosis being a complication of diabetes, both conditions have common genetic and environmental antecedents, i.e., they spring from a “common soil”. He also stated that “fetal and early life are associated with an enhanced risk of both diabetes and cardiovascular disease many decades later”, and “the same adverse environmental conditions are also associated with the development in adult life of abdominal obesity and the insulin-resistance syndrome” [5].

“Common soil” hypothesis for T2D and CVD is relevant even today, 25 years after it was presented. Recently published largescale association studies and especially MR studies have produced new information that helps to understand the relationship between T2D and CVD. These studies showed that T2D is a causal risk factor for CVD, but CVD is not a causal risk factor for T2D, although very often these diseases occur in the same individuals. As predicted by Stern, low birth weight is a causal risk factor for both T2D and CVD.

Obesity, overweight, and abdominal obesity are drivers for the epidemic of T2D. MR studies reported that increased BMI and waist-to-hip ratio are causal not only of T2D but also of CVD. Furthermore, MR studies showed that increased blood pressure, smoking, elevated total triglycerides, and ALT concentrations are causal risk factors for CVD and T2D. This shows that there are several risk factors shared by T2D and CVD, and, therefore, these diseases share “common soil”. However, there are differences between T2D and CAD with respect to inflammatory markers and LDL cholesterol.

Low-grade inflammation plays an important role in the development of T2D and CVD. MR studies showed that IL6 and CRP are causal for the development of atherosclerosis and CAD, but there are no studies on the role of IL6 as a risk factor for T2D, and CRP has not been demonstrated to be causal for the risk of T2D. Based on the current knowledge, the major difference in the risk factors for T2D and CVD is LDL cholesterol. According to MR studies, elevated LDL cholesterol is an important causal risk factor for CVD, but LDL cholesterol levels tend to be lower in patients with T2D.

The risk for both T2D and CVD is determined by lifestyle factors and genetic background. Major differences exist in the genetic background between these two diseases. Importantly, only a few genetic loci jointly contribute to the risk of T2D and CVD. Estimates of the heritability of T2D vary widely around a median of 40%, suggesting that around half the genetic contribution to the variation in risk can be quantified. If the estimates of relative risk observed in UK Biobank participants generalize to the population level, then these individuals have about 50% lifetime risk of T2D [107]. In contrast, genetic variants from GWAS studies explain only about 20% of the genetic risk of CAD [108].

In conclusion, based on MR analyses including genetics, microbiota, and metabolomics, “common soil hypothesis” holds for almost all risk factors for T2D and CAD, but the roles of low-grade inflammation and LDL cholesterol are different between these two diseases.

## 11. Future Perspectives

“Common soil hypothesis” has been a relevant concept for clinical medicine by emphasizing the clustering of risk factors in individuals at a high risk of T2D and CVD. However, there is a need for additional studies. The mechanisms increasing the risk of T2D and CVD are still poorly known; therefore, further studies are needed to identify new pathways for these two diseases. Metabolomics studies have been often too small, including only a small number of participants and/or metabolites, and have not had a long follow-up period. Therefore, more large-scale prospective metabolomics studies are needed to identify metabolites from different pathways as risk markers for T2D and CVD. Currently, only a few MR studies have been published about the significance of metabolites as biomarkers for T2D or CVD. Large scale metabolomics studies are needed, especially on different ethnicities, since most of the studies published included only white Europeans, although almost 80% of adults with diabetes are living in low- and middle-income countries [1]. MR analyses have several assumptions and limitations which should be considered when making clinical implications from these studies.

In recent years, several studies have indicated that T2D is a heterogenous disease [103,104,105,106]. Several subgroups have been identified in T2D, which are associated with different pathologies and distinct clinical outcomes, including hypertension, CAD, and stroke [103]. Thus, it is likely that “common soil hypothesis” does not apply to all subgroups of T2D. CVD is an even more heterogenous disease, especially with respect to the genetic background, and it is not likely that this disease can be classified into subgroups in the near future.

## Figures and Tables

**Table 1:** Mendelian randomization studies on the risk factors for type 2 diabetes and cardiovascular disease.

		Type 2 Diabetes			Cardiovascular Disease		
Exposure	Genetic Instrument	Cases/Controls	Causal Effect Size or (95% CI)	Reference	Cases/Controls	Causal Effect Size or (95% CI)	Reference
Birth weight	PRS	3627/12,974	2.94 (1.70–5.16)	[68]	5542 /237,631	0.69 (0.60–0.80)	[69]
BMI	PRS	12,171/56,862	1.26 (1.17–1.34)	[70]	60,801/123,504	1.49 (1.39–1.60)	[71]
Waist:hip ratio	PRS	34,840/149,821	1.82 (1.38–2.42)	[72]	60,801/123,504	1.54 (1.38–1.71)	[71]
Blood pressure	PRS	37,293/125,686	1.02 (1.01–1.03)	[73]	255,714/10 mmHg increase	1.49 (1.38–1.63)	[74]
Smoking	PRS	74,124/824,006	1.28 (1.20–1.37)	[75]	60,801/123,504	1.48 (1.25–1.75)	[76]
LDLC	PRS	33,323/418,610	0.82 (0.63–1.00)	[77]	9647/451,933	1.44 (1.42–1.47)	[77]
Total TG	PRS	33,323/418,610	1.35 (1.19–1.51)	[77]	9647/451,933	1.31 (1.24–1.38)	[77]
ALT	PRS	33,323/418,610	1.73 (1.52–1.94)	[77]	9647/451,933	1.45 (1.10–1.92)	[77]
CRP	PRS	33,323/418,610	0.92 (0.76–1.09)	[77]	9647/451,933	1.12 (1.10–1.15)	[77]
IL-6	PRS	Not available	−	−	9417/15982	1.60 (1.60–1.72)	[78]
Apo-B	PRS	Not available	−	−	60,801/123,504	1.73 (1.56–1.91)	[79]

Abbreviations: ALT, alanine aminotransferase; APO-B, apolipoprotein B; BMI, body mass index; CRP, C-reactive protein; IL-6, interleukin 6; LDLC, low density lipoprotein cholesterol; TG, triglycerides.
